# The Trade-Off between Airborne Pandemic Control and Energy Consumption Using Air Ventilation Solutions

**DOI:** 10.3390/s22228594

**Published:** 2022-11-08

**Authors:** Ariel Alexi, Ariel Rosenfeld, Teddy Lazebnik

**Affiliations:** 1 Department of Information Science, Bar-Ilan University, Ramat-Gan 5290002, Israel; 2 Department of Cancer Biology, Cancer Institute, University College London, London WC1E 6DD, UK

**Keywords:** airborne transmission and control, indoors pandemic spread, air ventilation, energy consumption

## Abstract

Airborne diseases cause high mortality and adverse socioeconomic consequences. Due to urbanization, more people spend more time indoors. According to recent research, air ventilation reduces long-range airborne transmission in indoor settings. However, air ventilation solutions often incur significant energy costs and ecological footprints. The trade-offs between energy consumption and pandemic control indoors have not yet been thoroughly analyzed. In this work, we use advanced sensors to monitor the energy consumption and pandemic control capabilities of an air-conditioning system, a pedestal fan, and an open window in hospital rooms, classrooms, and conference rooms. A simulation of an indoor airborne pandemic spread of Coronavirus (COVID-19) is used to analyze the Pareto front. For the three examined room types, the Pareto front consists of all three air ventilation solutions, with some ventilation configurations demonstrating significant inefficiencies. Specifically, air-conditioning is found to be efficient only at a very high energy cost and fans seem to pose a reasonable alternative. To conclude, a more informed ventilation policy can bring about a more desirable compromise between energy consumption and pandemic spread control.

## 1. Introduction

Large-scale pandemic outbreaks that occurred in the last several decades such as the Hong Kong flu (Influenza A/H3N2), HIV/AIDS, and the coronavirus (COVID-19) pandemics are associated with wide-reaching economical damage, social disaster, and high mortality [1,2,3]. Wu et al. [4] suggested that the globalization processes that transpired in the last century have invigorated new pandemic outbreaks [5]. In particular, urbanization in the developing world is bringing more people into denser neighborhoods, which results in a higher infection rate, at which new diseases spread [6,7]. Indeed, the world’s population is concentrated in cities, and megalopolises in particular [8], which keep growing over time [9]. In addition, most individuals who live in cities spend most of their time indoors, mainly in building (e.g., home, school and workplace) [10].

In parallel, a global sharp increase in energy and goods consumption, associated with the increase in population size and life quality [11,12,13], has led to growing recognition for the importance of sustainable consumption in order to minimize costs and, most importantly, protect the planet from degradation so that it can support the needs of the present and future generations [14]. Indeed, in 2015, all UN member countries have adopted the 2030 Agenda for Sustainable Development [15] thus committing to making fundamental changes in the way that our societies produce and consume goods and services. In addition, the UN has highlighted the need to Ensure healthy lives and promote well-being as one of its core 17 sustainable development goals. In the context of pandemic control, minimizing energy consumption and reducing the spread of a pandemic are, in fact, partially conflicting goals [16,17]. Most importantly, in indoors settings, policymakers should consider pandemic control through a multi-objective perspective where both the pandemic spread as well as the energy consumption of the implemented measures are considered together. Indoor settings often provide fruitful grounds for the integration of sensors which can facilitate a real-time system to pursue its desired objective. In our context, sensors are an obvious candidate for measuring key measures associated with a pandemic spread and energy consumption [18].

A large body of work has focused on a broad spectrum of pandemic intervention policies (PIPs) such as lockdown [19,20], mask-wearing [21], social distance [22], inspection units [23], and vaccination [24] for airborne pandemics. Focusing on PIP for indoor settings, Lazebnik and Alexi [25] analyzed the spread of an airborne pandemic indoors in four types of buildings (home, school, office, and mall) while implementing different PIPs, showing significantly different spread patterns between them. Wei and Li [26] review the release, transport, and exposure of expiratory droplets in a close environment such as a room. The authors concluded that droplets or droplet nuclei are transported by airflow and suggested that face masks, as well as air ventilation, can significantly reduce the infection rate. Peng et al. [27] explored the pandemic spread of the COVID-19 pathogen for indoor settings using a spatio-temporal version of the Wells-Riley models [28]. The authors provided a formula for the connection between population density and pandemic spread. They show that mask-wearing is partially effective against air-borne pandemics in a close room, which a decreasing effectiveness over time. These and similar PIPs have shown promising results in controlling airborne pandemic spreads in both theory and practice [29,30]. The main limitation associated with most PIPs are their dependency on the willingness of the population to comply with them [31]. Historical records reveal that, indeed, populations across the globe are only partially complaint with PIPs even if these are formally regulated and enforced [32,33].

Promisingly, implementing air ventilation solutions such as air-conditioning have shown to be an effective PIP in mitigating the spread of pandemics in indoor settings [34]. In addition to the proved reduction in airborne transmission, their effectiveness does not require any active participation from the occupants of the ventilated space. Dai and Zhao [35] estimated the association between the infection probability and changes per hour of air in the room (i.e., ventilation rate), obtaining that to in order to ensure an infection probability of less than one percent, a ventilation rate larger than the regularly practiced in the US is required. Memarzadeh and Xu [36] showed that while the ventilation rate is important in reducing the pandemic spread, the dominant factor that affects the transmission and control of contaminants in closed rooms is the path between the contaminant source and exhaust. D’Orzio et al. [37] investigated the COVID-19 spread in university buildings, extending the analysis from a single room to a larger, more realistic, indoor environment. The authors used an agent-based simulation, relying on historical records, and obtained that standard air ventilation is not able to constrain pandemic outbreaks such as the COVID-19 pandemic without the implementation of additional PIPs, such as masks. However, the authors further suggested that a higher level of ventilation will allow less reliance on masks.

Naturally, controlling a pandemic using any air ventilation solution without explicitly considering its associated costs could bring about an unwarranted economical and ecological burden. On the other hand, considering only the latter would likely entail poor pandemic control as well. To the best of our knowledge, to date, no study has examined this multi-faceted challenge of controlling pandemic spread indoors using air ventilation solutions while accounting for the potentially significant energetic costs associated with them. We present the Pareto front of using three standard air ventilation solutions: open window, air-conditioning and a pedestal fan, which we study across three common room types: inpatient room, classroom, and conference room. The Pareto front, which consists of all Pareto efficient solutions, allows us to explore the trade-off within this set and identify potential inefficient solutions and configurations which rationally should be avoided. Thus, the novelty of the proposed work lies in the investigation of the energy consumption and pandemic spread control of several (well-distributed) air ventilation solution in several types of rooms using sensors and in silico pandemic spread simulation.

The paper is organized as follows: Section 2 describes the study design. Section 3 reports the results obtained in this study which are then discussed in Section 4. Future work directions are highlighted as well.

## 2. Methods

**Figure 1:**
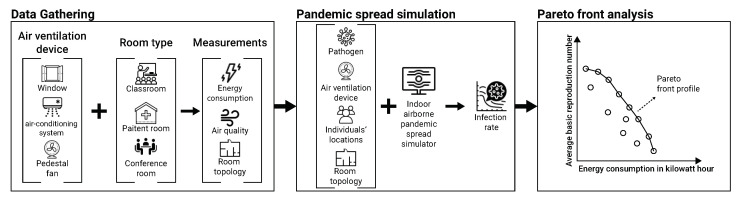
A schematic view of the research’s design, divided into data gathering, pandemic spread simulation, and epidemiological-energy Pareto front analysis. During the data gathering phase, we used several air quality and ventilation measurements alongside energy consumption to collect the required data for the pandemic spread analysis. Using the collected data, we estimate the average pandemic spread reduction using each ventilation solution via simulation. Finally, we perform a Pareto front analysis to study the trade-off between pandemic control and energy consumption and identify possible inefficient configurations.

In this study, we consider three standard air ventilation solutions: open window, an air-conditioning system and a pedestal fan which are studied across three room types: inpatient room, classroom and conference room. Overall, nine air ventilation to room type crossovers are considered in this work. We follow a simple three-phased study design consisted of in-the-field data collection, using a set of sensors, pandemic spread simulation, and epidemiological-energy Pareto-front analysis, as schematically presented in Figure 1. Each phase is discussed in detail below.

### 2.1. Data Collection

We first collected key measures for each of the nine crossovers in question. Starting with the room types, in order to ensure the credibility of our measures, three separate yet highly similar and closely located rooms were selected as representative for each type. For inpatient rooms, we used three 3 by 6 Meters inpatient rooms from one of the internal wards of Sheba Hospital (Ramat-Gan, Israel). Per Israeli regulations, all three inpatient rooms were occupied by 2 to 4 patients. Three roughly 8 by 8 Meters elementary school classrooms were further selected (Tel-Aviv, Israel) as representatives, each occupied by 31 to 36 pupils and a teacher. For conference rooms, we used three rectangle-shaped rooms from a modern office building (Ramat-Gan, Israel). The rooms slightly varied in size from 30 m${}^{2}$ to 40 m${}^{2}$ and were occupied by 6 to 11 individuals. All rooms were scanned using a 3D LiDAR-based scanning app. Namely, the *RoomScan LiDAR* application on an iPhone 12 device, which captured the room’s topology and occupants’ locations [38].

Considering the air ventilation solutions, each of the nine rooms had a window to the outdoors with a surface area of 6 by 11 m. The window was either closed (i.e., inactive) or completely open (i.e., active). Each room further had a modern, high quality and well-maintained air-conditioning system which was directed roughly to the center of the room. Since we are not interested in artificially changing the room’s temperature (see further discussion in Section 4), when activated, the air-conditioning system was set to the room’s current temperature and the blower was set to either low, medium or high speed. Similarly, a high quality, out-of-the-box pedestal fan, roughly pointing to the center of the room, was set to either low, medium or high speed when activated.

The key measures in our data collection, which are essential for simulating a pandemic spread as discussed next, are the room’s temperature, humidity, air quality, and associated energy consumption over time. The room temperature, humidity and air quality were monitored using the *Air Quality Monitor/Recorder, model PM-1064SD* device mounted at the center of the room. The energy consumption over time was recorded by a *Poniie-PN2000* device which was directly connected to the power supply.

The data collection in all room types were conducted in parallel in August 2022 in order to avoid external confounds. Before any air ventilation solution was activated, the air quality was sampled over 30 min in order to capture a baseline. Next, one of the systems was activated for 90 min, followed by a 60 min break, thus allowing the room to roughly return to the baseline condition. The process is repeated three time in total, one for each of the examined systems. The order at which the systems were tested was chosen at random. During the measurements, the occupants were asked to behave normally, yet avoid leaving the room or opening doors or windows. During the data recording, little to no movement by the occupants was detected. This may be attributed the functionality of the selected room types and population (i.e., hospitalized patients in a hospital room) which, unlike the occupants of a restaurant or an airport, present more static behavior.

### 2.2. Pandemic Spread Simulation

Measuring the pandemic spread directly is impossible as it would involve the intentional infection of individuals with a pathogen. Instead, we used a computer simulation to estimate the pandemic spread over time from the measured data collected by our sensors [39]. Specifically, we used the standard model proposed by [34]. This model uses a spatio-temporal simulation approach combining agent-based simulation [40] with breathing dynamics and a Susceptible-Exposed-Infected (SEI) epidemiological model [41] in addition to a Computational Fluid Dynamics (CFD) [42] for the air movement in a room. In particular, the model accepts a spatial configuration in the form of the room’s topology, the average locations of the individuals in the room, and the location and average ventilation rate of an air ventilation solution. In addition, the model accepts a temporal configuration in the form of the room’s temperature, and the pathogen’s basic infection and recovery rates. We provide the model with the room’s topology using the aforementioned LiDAR-based scanning. Moreover, the average locations of the individuals in the room were introduced manually based on the sitting/laying locations the individuals spent most of their time in the room. The average ventilation rate of the air ventilation solution is fitted to the airspeed data obtained during the experiments, using the gradient descent optimization algorithm [43] while taking into consideration the relative location of the device in the room and its average air suction force as reported by the manufacturer. For the epidemiological parameters required by the model, we used the COVID-19 pathogen (the *Alpha* variant) as it is the most recorded pandemic in history [44]. Specifically, we follow the values proposed by [34]. The temperature over time is provided to the model as well based on our measurements.

The model adopted in this work (which based on [34]) is constructed around three main components: a population of individuals, an environment (room) representing a three-dimensional space, in which the population is located, and air flow dynamics. The simulation is working in discrete steps over time. Initially, the simulation is used with the space’s geometry as obtained from the LiDAR scannings in the form of a dot cloud. In addition, the individuals’ locations are set manually, assuming they present little to no movement in the environment (as is the case in our data). Specifically, the location of the individuals’ faces are set to be 1.2 m above the floor and oriented approximately according to where they were looking. The simulation is assuming the individuals are breathing normally. We adopted the breathing dynamics as suggested in the original model. Afterwards, in each step in time, five actions take place: First, based on the individuals’ stage in the breathing cycle, a force is allocated to the location in which their faces are. In particular, infected individuals generate pathogen particles into the air. In a similar manner, the average air flow of each air ventilation solution is treated as a constant air flow force. Second, an adaptive mesh for the CFD model is computed [45]. Third, the air movement in a single step in time is computed using the RANS method [46] for the CFD model. Fourth, the number of pathogen particles that enter individuals’ bodies are updated. Finally, the epidemiological state of each individual in the population is updated based on the number of pathogen particles in the individuals’ bodies and their current epidemiological state, following the classic SEI model. This process repeats for *T* = 54,000 time steps in order to obtain 10 simulations steps each second for the 90 min of experiment. This value is aimed at balancing the simulation’s accuracy, that increases as the number of steps per second increases, and the computational demand that increases as well.

### 2.3. Pareto front Analysis

We computed the Pareto front of the energy consumption and pandemic spread reduction for each solution in question and room type using Matlab’s *multiobjective optimization* tool (version 2020a) [47] with the average basic reproduction number ($R_{0}$) [48] as the epidemiological signal and the average energy consumption of the implemented solution across the three samples as the energy consumption signal. In particular, we use the “gamultiobj” library that implementing the genetic algorithm [49,50] that can search for a globally optimal solution under given constrains as required to obtain the Pareto frontier. The statistical significance level was set at (*p* < 0.05).

## 3. Results

**Figure 2:**
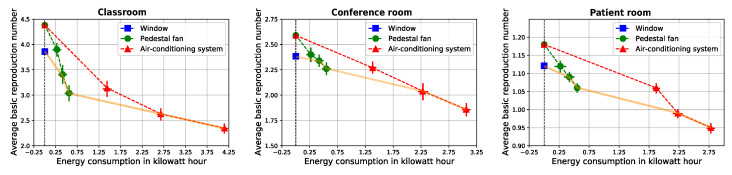
The energetic-epidemiological profiles for open window (blue square), pedestal fan (green dots), and air-conditioning system (red triangles) across three room types. The results shown as average ± standard deviation over 90 min. The x-axis indicates the average energy consumption in kilowatt hour of the air ventilation solution and the y-axis indicates the average basic reproduction number. The dashed black vertical line indicates no energy consumption. The solid orange line is the Pareto front.

Figure 2 displays the pandemic spread and energy consumption associated with each of the examined air ventilation solutions and room types where the x-axis is the energy consumption in kilowatt hour ([$10^{3}$ wh]) and the y-axis is the average basic reproduction number ($E[R_{0}]$). Each point indicates a 90-minutes experiment. The Pareto front is identified and highlighted in orange.

Starting with the open window condition, it significantly reduces the pandemic spread compared to the baseline of no air ventilation at all. In that respect, our simulation replicates a well-known result [51]. Naturally, open window has no associated energetic cost and thus it is depicted in Figure 2 as a single point along the dashed horizontal line. Interestingly, using the pedestal fan with a low fan speed is dominated by the open window condition for all three room types and thus it is not part of the Pareto front. Similarly, for all three room types, using the air-conditioning system with a low blower speed is dominated by the pedestal fan with a high fan speed, thus it is not part of the front either. The four remaining combinations (pedestal fan with either medium or high fan speed and air-conditioning with either a medium or high blower speed) are, in fact, efficient, with the fan-based outcomes presenting significantly lower energy consumption and higher pandemic spread compared to air-conditioning. In other words, for all three room types, the Pareto front consists of all three examined air ventilation solutions but not all their respective configurations.

## 4. Discussion

For all examined room types, the Pareto front consists of all three examined air ventilation solutions. By itself, this result means that none of the examined solutions should be a priori preferred or excluded from consideration, as at least one of its configurations is efficient. However, the results further demonstrate several significantly inefficient configurations as well. For example, using an air-conditioning system with low speed blower, one is expected to waste 2.5 to 4 times more energy compared to a pedestal fan at high fan speed, while resulting in even higher pandemic spread. Obviously, inefficient configurations are best to be avoided.

Deciding which of the efficient air ventilation configurations to implement in practice is a personalized task, one which depends on the decision-maker’s preference, budget and various other human factors (see [52] for an overview). Nevertheless, air-conditioning systems seem to be efficient only at very high energetic costs. Given that air-conditioning systems are also expensive and operationally challenging to install and modify in existing buildings, fans seem to offer a reasonable alternative. In many cases, and especially ones with limited budget, we believe that fans can strike a more desirable compromise between energy consumption and pandemic control. However, in cases where altering an existing air ventilation solution is challenging or impossible, policymakers can still take advantage of the identified trade-off profile between pandemic control and energy consumption of the given air ventilation solution to make a more informed decision. Taken jointly, the proposed work provides a first analysis of the trade-off between energy consumption and pandemic spread control for well-distributed air ventilation solutions across important room types. A natural conclusion one can draw from our results is that, while implementing some PIPs such as social distancing is laborious and restrictive, implementing air ventilation solutions to control for pandemic spread is relatively accessible, easy to monitor, and widely distributed even before an airborne pandemic begins.

It is important to note that our study does not consider additional factors which may influence the decision to adopt and configure an air ventilation solution in a certain way (see [53,54] for more details). For example, thermal comfort may play an important role as some may be more or less willing than others to reduce their energy consumption at the expense of some thermal discomfort [55]. Since thermal comfort is extremely hard to obtain, and highly personal in nature, we leave its investigation for future work. It is important to note that thermal comfort is highly interwoven with the energy cost and the pandemic spread. Specifically, the environmental conditions in a room such as temperature and humidity, which are accounted for in our simulation (see Section 2.2), have a direct biophysical influence on the pathogen’s particles and the pandemic spread [56]. As such, in contrast to the natural negative association between energy consumption and pandemic control identified here, there are presumably more complex and non-trivial connections that link the thermal comfort to the energy consumption and pandemic control which offer a fruitful avenue for future work. In addition, since modern pandemics show high mutation rate [57,58], a natural extension of this work is to evaluate the effectiveness of the examined air ventilation solutions on multi-strain pandemic settings by replacing the SEI model used in the simulation with multi-strain epidemiological models such as [59,60,61]. Moreover, as room-level air ventilation policy can be implemented in many cases, an additional promising research direction would be to find the optimal air ventilation type, location and configuration for each room given its topology, occupants, use patterns, etc. One can obtain a dynamic and room-type tailored policy using relatively cheap and easy to install sensors, such as those used in this work, thus using the proposed methodology seems to be appropriate [18,62,63].

## Data Availability

The data that support the findings of this study are available on request from the corresponding author.
